# Bioinformatic strategies for the analysis of genomic aberrations detected by targeted NGS panels with clinical application

**DOI:** 10.7717/peerj.10897

**Published:** 2021-03-31

**Authors:** Jakub Hynst, Veronika Navrkalova, Karol Pal, Sarka Pospisilova

**Affiliations:** 1 Center of Molecular Medicine, Central European Institute of Technology, Masaryk University, Brno, Czech Republic; 2 Department of Internal Medicine-Hematology and Oncology, Faculty of Medicine and University Hospital Brno, Masaryk University, Brno, Czech Republic; 3 Department of Medical Genetics and Genomics, Faculty of Medicine and University Hospital Brno, Masaryk University, Brno, Czech Republic; 4 Department of Hematology, University Hospital Schleswig-Holstein, Kiel, Germany

**Keywords:** Bioinformatic analysis, SNV/indel, CNV, Clinical application, Molecular markers, NGS, Targeted panels

## Abstract

Molecular profiling of tumor samples has acquired importance in cancer research, but currently also plays an important role in the clinical management of cancer patients. Rapid identification of genomic aberrations improves diagnosis, prognosis and effective therapy selection. This can be attributed mainly to the development of next-generation sequencing (NGS) methods, especially targeted DNA panels. Such panels enable a relatively inexpensive and rapid analysis of various aberrations with clinical impact specific to particular diagnoses. In this review, we discuss the experimental approaches and bioinformatic strategies available for the development of an NGS panel for a reliable analysis of selected biomarkers. Compliance with defined analytical steps is crucial to ensure accurate and reproducible results. In addition, a careful validation procedure has to be performed before the application of NGS targeted assays in routine clinical practice. With more focus on bioinformatics, we emphasize the need for thorough pipeline validation and management in relation to the particular experimental setting as an integral part of the NGS method establishment. A robust and reproducible bioinformatic analysis running on powerful machines is essential for proper detection of genomic variants in clinical settings since distinguishing between experimental noise and real biological variants is fundamental. This review summarizes state-of-the-art bioinformatic solutions for careful detection of the SNV/Indels and CNVs for targeted sequencing resulting in translation of sequencing data into clinically relevant information. Finally, we share our experience with the development of a custom targeted NGS panel for an integrated analysis of biomarkers in lymphoproliferative disorders.

## Introduction

Recent progress and growing application use of next-generation sequencing (NGS) technology, alongside the reduction of costs, has revealed new prospects in the field of personalized medicine. Researchers and clinical laboratories worldwide are implementing NGS to identify defects in the cancer genome to improve patient stratification and treatment. These genomic aberrations are represented by single nucleotide variants (SNVs), small insertions and deletions (Indels), copy number variants (CNVs) and structural variants (SVs), which accumulate in the genome during tumor development. Some of them are present at the time of diagnosis, while others occur as a consequence of clonal evolution during the disease course (Wang et al., 2014; Landau et al., 2015). Over the past years, it has been demonstrated that NGS is a unique tool for identifying new genomic variants (Armaou et al., 2009; Ascierto et al., 2012; Lindsley et al., 2015; Zoi & Cross, 2015), which can serve as important diagnostic and prognostic markers in various cancer types. In the field of hematooncology, rapid adoption of NGS had an enormous influence on the understanding of the genetic landscape, clonal evolution and prognostic impact of new molecular markers during the disease course (Landau et al., 2015; Pastore et al., 2015; Nadeu et al., 2016; Papaemmanuil et al., 2016; Dubois et al., 2016).

An expanding catalogue of molecular markers with clinical importance can be analyzed by targeted DNA sequencing of relevant regions in a fast, effective, and accessible manner (Paasinen-Sohns et al., 2017). While targeted sequencing enables the detection of various alterations in recurrently affected genomic regions, alternative NGS techniques, such as whole-exome sequencing (WES) or whole-genome sequencing, can be used to identify additional disease-related markers outside the areas of targeted assays (especially genome-wide CNVs and SVs). Nevertheless, a higher cost and an overwhelming amount of produced data (Metzker, 2010) requiring extensive and time-consuming bioinformatic analysis limit their usage in routine diagnostics (Kuo et al., 2017). The use of targeted NGS panels has indeed proven to be a financially feasible approach, providing benefits in cancer patient management (Hamblin et al., 2017). Moreover, larger targeted panels (>1 Mb) with the adjustments of design and cutoff values (Allgäuer et al., 2018; Heydt et al., 2020) can nowadays substitute the use of WES for the estimation of tumor mutation burden, which serves as a surrogate predictive marker in cancer (Rizvi et al., 2015). In general, a variety of commercial cancer-specific panels is available and widely used to detect genomic changes in different cancer types (Nikiforova et al., 2018; Steward et al., 2019). However, an off-the-shelf approach may not always be appropriate for clinical use as these panels may include several genes without established clinical importance (e.g., genes investigated in clinical trials or research studies) or may lack other genes of interest. A trend towards customization of targeted panels to fulfil the needs of individual laboratories is evident. The decision whether to use a commercial NGS panel or whether to put effort and labor into the design, validation and development of a respective bioinformatic pipeline strongly depends on the clinical utility, available bioinformatic support and financial and time capabilities of individual laboratories.

The implementation of a reliable bioinformatic pipeline developed with respect to the experimental approach is a major challenge for many clinical laboratories. Although some targeted panels have been published together with their tailored pipelines (Kluk et al., 2016; Soukupova et al., 2018), additional validation is necessary for each pipeline to confirm or adjust panel specifics including the accuracy and sensitivity. The process of tailored pipeline development starts with comprehensive literature and software survey followed by an in-depth evaluation of results produced by every single bioinformatic step. Despite several published best practice bioinformatic guidelines (Van der Auwera et al., 2013; Gargis et al., 2015), the development of a pipeline is still a time-consuming and laborious procedure. The resulting pipeline has to be highly reliable, adjusted to specific laboratory needs and flexible to demands changing over time.

In this review, we discuss the possibilities of a custom targeted NGS panel implementation with a particular focus on bioinformatics. We emphasize state-of-the-art approaches for the identification of genomic aberrations from DNA NGS data to make the process of bioinformatic pipeline development more transparent. Finally, we share our experience with the evaluation of a custom panel designed for a comprehensive analysis of genomic markers in lymphoproliferative disorders with the emphasis on bioinformatic tools assuring accurate results.

## Review methodology

The motivation behind the compilation of this review was our hands-on experience with the implementation of a custom targeted NGS panel for lymphoproliferative disorders and the scarcity of bioinformatic publications accompanied by practical experience in the development of specific bioinformatic pipelines in clinical use. Relevant and highly impacted articles from 2009 to the present, spanning the field of bioinformatics, cancer genomics and hematooncology, were scrutinized and systematically reviewed. The bibliography was created using the Zotero citation manager.

## Experimental design remarks

During the design of a custom targeted NGS panel, issues such as intended use, panel size with respect to anticipated coverage, clinical validity and utility must be considered. Each laboratory should think over its facilities, time and cost demands, sample turnaround, flexibility and bioinformatic support. It is also essential to determine the spectrum of targeted aberrations before selecting a target enrichment approach and an NGS platform. Generally, targeted NGS gene panels are designed to identify variants such as SNV/Indels and are either limited to only well-described hotspot mutations in clinically relevant genes (especially in routine diagnostics) or include whole coding sequences and splice sites. In addition, larger panels may target selected CNVs and SVs requiring more sophisticated bioinformatic analyses. For the detection of subclonal aberrations, a high sequencing depth is necessary. Crucial recommendations for NGS panel and bioinformatics pipeline validation were published by the Association for Molecular Pathology and College of American pathologists (Jennings et al., 2017; Roy et al., 2018).

**Figure 1:**
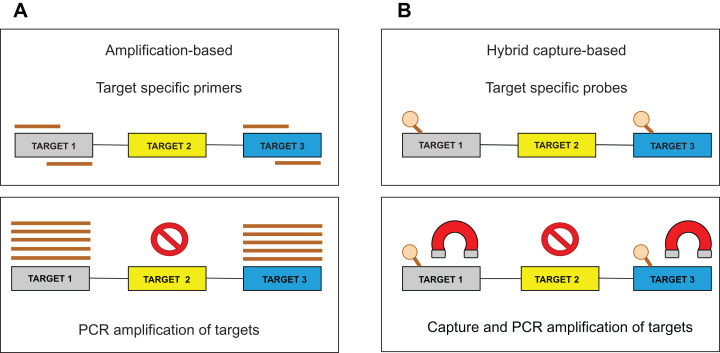
Target enrichment approaches for NGS library preparation. (A) The amplification-based method with the use of the PCR primers. (B) The hybrid capture-based method utilizing target-specific probes.

Target enrichment is an essential step in NGS testing. For sequencing library preparation, two major approaches are used: (1) hybrid capture-based and (2) amplification-based (Fig. 1). Several studies describe the benefits and drawbacks of both methods (Sulonen et al., 2011; Chilamakuri et al., 2014; Gargis et al., 2015; Kuo et al., 2017). The amplification approach is based on the multiplex polymerase chain reaction (PCR) method, which requires short hands-on time, but also precise PCR optimization to produce uniform amplification efficiency across all targets. Moreover, PCR amplification is less efficient in regions with high guanine-cytosine (GC) content or in repetitive regions. Importantly, the identification of larger Indels or chromosomal rearrangements is generally complicated since these aberrations could span over the location of a PCR primer. In the hybrid capture approach, sequence-specific probes are designed to catch DNA fragments of interest. These biotinylated oligonucleotides are significantly longer than PCR primers and can, therefore, tolerate the presence of several mismatches resulting in an effective enrichment process. Generally, it has been shown that the capture-based methods show better performance than amplification-based ones, with respect to sequencing complexity and uniformity (Samorodnitsky et al., 2015). Besides, the amount of captured DNA is proportional to the DNA present in a sample allowing CNV detection by a “read depth” approach. The method is also less affected by DNA quality, enabling the analysis of such biological materials as formalin-fixed, paraffin-embedded (FFPE) blocks (Hung et al., 2018) or circulating free DNA (cfDNA) (Rossi et al., 2017). Several commercial targeted NGS technologies are available in custom design and their comparison performed by Samorodnitsky et al. (2015) could serve as an informed decision for individual laboratory applications.

**Figure 2:**
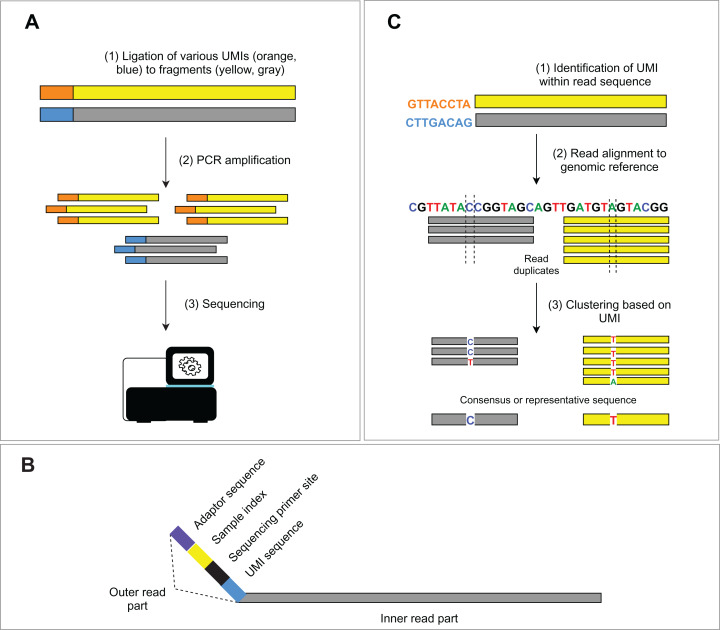
Schema of UMIs experimental and bioinformatic processing. (A) During NGS library preparation, UMIs are ligated to DNA fragments, followed by PCR amplification and sequencing. (B) Structure of the outer part of a read is depicted. (C) Bioinformatic read deduplication and error correction using UMI identification and read clustering. Depending on the selected tool, deduplication results either in a consensus read creation or the selection of the most representative read.

The occurrence of duplicated DNA fragments generated during the amplification step in library preparation represents a common issue in NGS data analysis. It is difficult to determine which sequences originate from genomic DNA and which are a product of PCR amplification. Unique molecular identifiers (UMIs) (usually 8–12 bp long), ligated to genomic fragments before the first PCR amplification, help solve this problem (Fig. 2A). Nonetheless, the incorporation of the UMIs increases the cost and uses up some of the assay capacity. The consequent bioinformatic analysis requires additional solutions to utilize UMIs in PCR duplicate recognition. Subsequent steps of deduplication and the creation of consensus sequences lead to the in silico removal of sequencing duplicates and thus increase the sensitivity of the assay. We discuss bioinformatic solutions for read deduplication with the use of UMIs in the bioinformatic part.

## Bioinformatic data analysis

### Bioinformatic workflow management

In any NGS experiment, a bioinformatic pipeline needs to fulfil several analytical requirements determined by the assay design. In other words, the analysis has to ensure the accuracy and reproducibility of results under individual experimental settings, which is especially relevant in routine diagnostics. Both properties are likewise essential when investigating the clonal expansion of aberrations across different time points during the disease course. Proper workflow management helps fulfil these requirements by ensuring software installation and versioning, organization of input and output data, and resource management during the execution of a pipeline.

Software (package) management can be ensured by the Conda package management system. Conda provides a unified method of software installation and concurrently allows the management of individual projects within specific environments, ensuring consistency and stability. The *Bioconda channel* (Grüning et al., 2018) specializes in tools used in bioinformatic analysis. The execution of several programs chained through their inputs and outputs is managed by workflow engines such as Snakemake (Köster & Rahmann, 2018), Bpipe (Sadedin, Pope & Oshlack, 2012), reflow (https://github.com/grailbio/reflow) and nextflow (Di Tommaso et al., 2017), which also offers a set of curated pipelines under the nf-core project (Ewels et al., 2020). The “chaining” provides efficient administration of input and output data while monitoring used processes and resources. The workflow engines are managed through text-based configuration files.

Alternatively, the open-source Galaxy framework (Afgan et al., 2018) provides a user-friendly graphical interface and thus caters to users with little or no prior programming experience. It contains a comprehensive repository of preinstalled software for genomic, proteomic, or metabolomic data processing, which can be easily managed and combined into an analytical pipeline. A public Galaxy server provides computational resources sufficient for experimenting and small analyses with more than 124,000 registered users worldwide.

Yet another approach is represented by the bcbio-nextgen project (Chapman et al., 2020), which offers a resource manager (for both tools and data resources) as well as a collection of curated, reusable, highly optimized pipelines. On top of this, pipeline execution is highly customizable through configuration files. This solution shifts a considerable part of the workload on the community behind the development and maintenance of bcbio and is suitable for common applications of NGS.

Finally, one of the most recent yet already widely used technologies are containers such as docker (Merkel, 2014) and singularity (Kurtzer, Sochat & Bauer, 2017). They allow for a self-contained environment, in which analyses can be run. More importantly, the benefit of encapsulation lies in the ability to distribute a given container, which significantly improves the reproducibility of analyses. Essentially, all current pipeline managers support containers, and containers are in exchange supported by a wide range of underlying architectures, including cloud services mentioned in the following chapter.

### Hardware requirements

Appropriate hardware infrastructure is required to perform bioinformatic data analysis. It is important to understand the demands on hardware equipment because insufficient capacity could potentially lead to poor or erroneous results (Wade, Curtis & Davenport, 2015). For targeted NGS panel data analysis in diagnostic use, the basic hardware essentials are: (1) sufficient space to store pipeline inputs and outputs including intermediate or temporary files, (2) enough computational capacity (number of CPUs/GPUs and RAM size) and importantly, (3) security for sensitive data management.

There are several external options, which fulfilled the above-mentioned criteria. Commercial Amazon Web Services’ Elastic Compute Cloud (Amazon, 2020) provides scalable computational and storage resource to perform a range of bioinformatics analyses. Non-commercial institutional clouds such as European Grid Infrastructure (EGI) (EGI, 2020) or US The Open Science Grid (OSG, 2020) provide free advance computing services for scientists and research infrastructures. However, it is often advantageous to administrate local hardware for better control over computational resources, data security, and software management. The scale of this computational facility for each lab performing NGS experiments is individual, depending on sample turnaround, the number of ongoing projects and previous experience. In the last chapter, we showcase and further discuss the measurements of the consumption of computational resources of an exemplary analysis of our custom NGS panel. In general, a minimal analytical pipeline, akin to the one presented here, consisting of only the essential steps (read alignment, variant calling and variant annotation) could potentially run on a laptop equipped with at least 8GB of RAM. As these crucial steps undergo the most scrutiny (Lenis & Senar, 2015), they are highly optimized for speed and memory efficiency. Despite this possibility, the general trends favor commercial/institutional cloud services and/or large computational servers optionally with dedicated hardware such as GPU’s and even FPGA’s. Several such GPU implementations for both the alignment and variant calling have been published (Klus et al., 2012; Cardoso et al., 2016; Ahmed et al., 2019; Ren et al., 2019).

### Sequencing reads preprocessing and alignment

Short, single or paired-end reads are produced by the Illumina sequencers, the most widely used sequencing platform. The read structure (Fig. 2B) consists of the inner part (the actual sequenced DNA fragment, so-called “insert”) and the outer part comprising a variable combination of: (1) platform-specific sequences for binding the fragment to a flow cell (adaptor sequences), (2) single or dual indexes for read assignment to an individual sample, (3) sequencing primer sites for paired-end reads and optionally (4) molecular barcodes (i.e., UMIs) used for tagging the individual DNA molecules in a given sample library. Adaptor sequences and indexes are unnecessary for downstream analysis and are removed from the beginning of the read. Trimming is done during the demultiplexing step, where sequencing base calls are transformed into text-based read representation and stored in FASTQ formatted files. The result of the demultiplexing step is usually a pair of FASTQ files (R1 and R2, representing forward and reverse read) per sample. A third FASTQ file (R3) may be generated to store UMI sequences or, optionally, UMIs may be written in the read names of the R1 and R2 files. Finally, parts of the sequencing adaptors may also be present at the end of a read (given a short insert) and can also be removed from the read structure of both read pairs. This step is debatable and may be considered irrelevant as the adaptor sequences will be “soft-clipped” by the aligner. However, several variant callers are “soft-clip aware” (e.g., GATK’s HaplotypeCaller and Mutect2) and will try to realign the sequence around the soft-clipped bases. We, therefore, find adaptor (as well as quality) trimming generally advisable. Recently, more than 30 adaptor trimming tools were published, including the popular Cutadapt (Martin, 2011), Trimmomatic (Bolger, Lohse & Usadel, 2014) or the fastp (Chen et al., 2018) program.

Consequent quality control (QC) of preprocessed reads ensures sequencing data quality and integrity. FASTQ files contain (apart from the actual sequences of reads) read quality information represented by Phred Quality score (Q); base-calling error probability per each base. The FastQC tool can be used to summarize and visualize the Phred Quality score, GC content, sequencing into adaptors, the occurrence of overrepresented sequences and other parameters. Unsatisfactory reads with low Q scores can be trimmed or discarded from further downstream analysis by most of the standard trimming tools.

The preprocessed FASTQ files are subsequently mapped to the reference genome during the alignment step. The most recent human genome assembly is currently available in two, mostly interchangeable, versions; the GRCh38 (Schneider et al., 2016) reference (managed by the Genome Reference Consortium) and the hg38 reference (managed by UCSC). An older version of the reference hg19 (GRCh37) (Church et al., 2011) is still widely used. In general, the alignment is a fundamental and computationally demanding step, which allows the mapping of a sample onto the reference sequence (i.e., assigning genomic coordinates to each read). This process is error-tolerant, as mismatches between the sequencing reads and the reference may represent genomic variability and, more importantly, real pathogenic variants. The result of the alignment step is stored in a Sequence Alignment Map file or its more compact binary counterpart (BAM file). Within this format, each read contains additional information about genomic alignment coordinates, mapping quality information (MAPQ), splice alignment indicator (Compact Idiosyncratic Gapped Alignment Report, CIGAR) and more. MAPQ is often used to identify low-quality reads, which may adversely affect the identification of gene variants. The CIGAR string is a compressed representation of the alignment of an individual read, encoding which parts of the read match or mismatch the genomic reference and whether there are inserted or removed bases. MAPQ and CIGAR are helpful indicators for proper detection of real genomic variants as well as for determining alignment bias, which arises mainly from sequencing errors or repetitive regions. Freely available software for DNA read alignment such as NovoAlign (Novocraft, 2020), Bowtie2 (Langmead & Salzberg, 2012), Smalt (SMALT: Wellcome Sanger Institute, 2020) and Stampy (Lunter & Goodson, 2011) can be used, but they provide non-consistent results in various datasets (Ruffalo, LaFramboise & Koyutürk, 2011). Burrows-Wheeler aligner (BWA) is currently the most common software used for mapping (Li & Durbin, 2009), showing the best performance across multiple NGS datasets in alignment sensitivity, computational time, or the alignment of reads in repetitive regions (Thankaswamy-Kosalai, Sen & Nookaew, 2017). Therefore, BWA represents the preferred alignment tool for the majority of DNA based NGS techniques, including targeted panel assays.

Sample specific BAM files serve as input for the consecutive analytical branches, including the detection of SNV/Indels, CNVs and SVs. Before these analyses, PCR duplicates should be marked and removed from the BAM files to increase the accuracy of testing. This step is essential for the detection of low frequent somatic SNV/Indels and CNVs. The need for duplicates removal is amplified when analyzing fragmented low-quality DNA samples, for example, FFPE. It was shown that the overall duplication level in FFPE samples reached ~50–60% compared to less than 20% for DNA from fresh frozen tissue (Bewicke-Copley et al., 2019). To remove PCR duplicates, two approaches can be applied: (1) Picard tool *mark duplicates* (https://broadinstitute.github.io/picard/) is used to either tag or remove duplicated reads according to identical alignment coordinates of sequencing reads, or (2) use of UMIs processed by designated bioinformatics tools described in the following chapter.

### UMI processing for in silico read deduplication

The employment of molecular barcodes for the labeling of different DNA fragments is not recent (Jabara et al., 2011; Liang et al., 2014) and is nowadays implemented in many NGS assays. This advanced experimental approach allows precise identification of PCR duplicates and sequencing error correction in silico. Specifically, dual indexing with UMIs resolves index swaps, increases the sensitivity of variant detection and reduces inaccuracies in read count-based analyses (e.g., gene expression analysis, CNV analysis) (MacConaill et al., 2018; Costello et al., 2018). Bioinformatic approaches for UMI processing mostly rely on read alignment and consist of three main steps (Fig. 2C): (1) UMIs are identified within the read structure, and corresponding read pairs (forward/reverse) are tagged with the sequence of the UMI, (2) position matched reads (i.e., reads mapping exactly to the identical location on the reference genome) sharing the same UMI are clustered into groups, (3) read clusters are then collapsed into a consensus sequence, or a representative sequence is selected. For example, UMI-tools (Smith, Heger & Sudbery, 2017) *dedup* approach uses the highest alignment quality (MAPQ) to choose the most representative read sequence, while the fgbio (fgbio, 2020) *CallMolecularConsensusReads* applies a likelihood model to each base of the source read molecule, which finally leads to a consensus read creation. Other commonly used software is gencore (Chen et al., 2019b) or Je (Girardot et al., 2016). Alternative approaches, like the Calib program (Orabi et al., 2019), utilize alignment-free clustering, which is more suitable for high coverage amplicon sequencing. Nevertheless, this approach does not take into account potential substitution errors in UMI sequences.

### Evaluation of target enrichment efficiency

Coverage analysis is used to assess the efficiency of the primers or probes, which helps with the optimization of the target enrichment process. It is also essential to evaluate the uniformity of coverage across targets and sequencing runs to ensure maximum result reproducibility. Further, the ability to detect any genomic variants, correctly estimate VAF, and to reduce the false-positive rate at the same time improves with increasing depth and coverage uniformity (Sims et al., 2014). The effect of deduplication directly translates into the decrease in coverage and can be observed by comparing coverage statistics before and after the removal of duplicates.

Should a more thorough examination of coverage, both on- and off-target, be required, bedtools (Quinlan & Hall, 2010) *coverage* provide per-base coverage information across the whole genome or specific regions specified within a BED file (Ensembl., 2020). Such comprehensive information can be used to get a table of statistics such as min/max read depth, mean or median across defined regions and thus enabling disclosure of probable off-target locations. Statistics regarding the distribution of aligned reads between the targeted and non-targeted regions are an important quality control metric of sequencing assay design. The proportion of on-target reads covering targeted genomic regions enables the assessment of enrichment efficiency. The assignment of roughly >70% of the reads to target regions is considered a good library indicator (Hung et al., 2018), but this strongly depends on panel size and overall depth of coverage. A lower amount of the on-target reads can indicate an abundant occurrence of repetitive or homologous sequences in the targeted regions resulting in poor enrichment.

### Variant identification algorithms

Bioinformatic approaches for genomic variant identification are aimed to distinguish true biological variants from sequencing background. Dedicated programs are in abundance and, therefore, it is essential to understand underlying algorithms and parameters controlling their behavior to select the correct tool. Independent analytical branches identify different types of aberrations. We will predominantly discuss approaches and software for the detection of somatic and germline SNVs/Indels and CNVs. Furthermore, we will discuss different analytical procedures according to the experimental settings, for example, the availability of matched control (non-tumor or “normal”) sample from the same individual. For SVs, specific probes or primers can be designed to enrich and detect those events (McConnell et al., 2020). We remark that the common division of CNVs and SVs to the distinct groups is debatable since SVs, in general, include quantitative CNVs (comprising deletions, insertions, and duplications), translocations and/or inversions (Scherer et al., 2007). However, alterations that do not change the copy number of the genome have to be detected by specific algorithms (Guan & Sung, 2016).

#### Variant calling of SNV/Indels

The general strategy for variant identification is the calculation of the proportion of non-reference bases in a batch of reads that cover each position in a targeted region. The analysis of germline variants is straightforward since their VAF is about 50% or 100%, and the level of sequencing noise is expected to be of lower frequencies. GATK *HaplotypeCaller* (McKenna et al., 2010), MAQ (Li, Ruan & Durbin, 2008) or inGAP (Qi et al., 2010) represent germline-only variant callers. Nevertheless, some somatic callers can identify both germline and somatic variants such as Strelka2 (Kim et al., 2018), Varscan2 (Koboldt et al., 2013) or Octopus (Cooke, Wedge & Lunter, 2018). Further, we focus on the analysis of the somatic SNV/Indels since they represent a more challenging task compared to the detection of germline variants.

In the case of somatic variant calling, it is necessary to distinguish minor variants from background noise because true somatic variants can occur at low frequencies, especially in samples with low purity or rare tumor subclones. Herein, a statistical evaluation is critical to determine the underlying genotype and to discriminate among variants and artefacts. An ideal scenario for precise somatic variant identification is a paired sample analysis, where tumor and matched normal sample are compared. Variants present in the non-tumor sample are considered germline and excluded from subsequent interpretation. Several studies have evaluated somatic variant calling with default parameters in a tumor/normal setting (Ellis et al., 2012; Parry et al., 2015; Chen et al., 2015). The most popular open-source variant callers (Table 1) used diverse strategies for variant filtering that leads to differently reported variants demonstrating low concordance among various calling algorithms (Liu et al., 2013; Ewing et al., 2015; Xu, 2018). Cacheiro et al., 2017 concluded that each evaluated caller exhibits a different ability to call SNV/Indels properly and showed discrepancies in the estimation of VAF. In a study by Bian et al., 2018, various callers like FreeBayes (Garrison & Marth, 2012), VarDict (Lai et al., 2016), GATK *MuTect*, GATK *Mutect2* (Benjamin et al., 2019) and MuSE (Fan et al., 2016) were tested to assess specificity and sensitivity. GATK *MuTect2* shown the best performance according to evaluated metrics, including the highest ratio of true/false positive variants across multiple datasets. (Chen et al., 2019a) showed that Strelka2 (Kim et al., 2018) identifies variants accurately and outperforms other tools in computational costs. The computational time of analysis is important, especially in routine diagnostics, where results need to be obtained quickly. Rapid end-to-end analysis of sequencing data contributes to the improvement of cancer patient management, particularly in terms of effective therapy initiation. Combining results of multiple somatic callers to get consensus calls is more time consuming but provides more accurate variant calling. However, taking the intersection or the union of the call sets can lead to a drop in sensitivity or an increase in false-positive variants, respectively (Callari et al., 2017).

**Table 1:** A list of commonly used open-source variant callers and their usage possibilities.

Variant caller	Tumor-only mode	Variant type detection	References
CaVEMan	NO	SNV	Jones et al. (2016)
DeepSNV	NO	SNV	Gerstung et al. (2012)
DeepVariant	YES	SNV, Indel	Poplin et al. (2018)
EBCall	NO	SNV, Indel	Shiraishi et al. (2013)
FreeBayes	YES	SNV, Indel	Garrison & Marth (2012)
HapMuc	YES	SNV, Indel	Usuyama et al. (2014)
LocHap	NO	SNV, Indel	Sengupta et al. (2016)
LoFreq	YES	SNV, Indel	Wilm et al. (2012)
MuSE	NO	SNV	Fan et al. (2016)
Mutect	YES	SNV	Cibulskis et al. (2013)
Mutect2	YES	SNV, Indel	Benjamin et al. (2019)
Octopus	YES	SNV, Indel	Cooke, Wedge & Lunter (2018)
Platypus	YES	SNV, Indel	Rimmer et al. (2014)
SAMtools	YES	SNV, Indel	Li et al. (2009)
SomaticSniper	NO	SNV	Larson et al. (2012)
Strelka2	NO	SNV, Indel	Kim et al. (2018)
UMI-VarCal	YES	SNV	Sater et al. (2020)
VarDict	YES	SNV, Indel	Lai et al. (2016)
VarScan2	YES	SNV, Indel	Koboldt et al. (2012)

A matched non-tumor sample is not always easily accessible, especially in routine diagnostics or in retrospective analyses. Fortunately, some callers allow a “tumor only” mode of variant calling (Table 1), albeit with some additional challenges. Such calling indispensably requires the filtering of germline variants according to the information available in public population databases. However, this approach is not comprehensive as each individual could potentially have unknown germline variants, which are not included in the databases and could be wrongly considered somatic (Jones et al., 2015). Efficient identification of relevant mutations also strongly depends on the sufficient read depth and exclusion of false-positive calls. Some tools for tumor-only variant calling try to solve the stated issue with machine learning (Kalatskaya et al., 2017), but this requires large training datasets, which are not usually available during the implementation of targeted panels. This approach also does not consider the dynamics of tumor clone development in time. The validation of discovered variants is highly recommended not only for in-house developed algorithms but also for all commercial or freely available components (Roy et al., 2018).

#### Variant annotation and prioritization

Most variant callers use Variant Call Format (VCF) files to collect identified variants. VCFs store information such as mutation genotype, variant frequency, or genomic coordinates. Accurate annotation of generated variants is crucial for subsequent interpretation. Generally, the process of variant annotation integrates genomic variants into a functional and clinical context. Recently, several tools such as variant effect predictor (McLaren et al., 2016), ANNOVAR (Wang, Li & Hakonarson, 2010), SnpEff (Cingolani et al., 2012) or GATK VariantAnnotator were developed to provide comprehensive annotations including variant classification, standardized nomenclature, functional prediction, and information from available databases (Table S1). For routine diagnostics, it is essential to adhere to the unified nomenclature proposed by the Human Genome Variation Society (HGVS) (Claustres et al., 2014), which is a standard in variant description used for clinical reports. Mutalyzer (Wildeman et al., 2008), a web-based software, can be used to retrieve proper HGVS nomenclature for particular variants. However, the variant description itself is not sufficient for the assessment of its functional and clinical impact in the majority of cases. Therefore, multiple clinically relevant annotations are provided by tools collecting information from population databases or reports in published literature, or, in some cases, the effect of a variant may be estimated in silico (Table S2). Such information is permanently updated and is accessible through mentioned stand-alone annotation tools, which can be easily implemented within a bioinformatic pipeline. Concurrently, the web-based Cancer Genome Interpreter (Tamborero et al., 2017) can be used to annotate somatic variants found in tumor samples, and it automatically predicts the possible role of a variant in tumorigenesis and treatment response.

After annotation, a prioritization of variants is performed to select clinically relevant variants to be reported to a clinician. Variant filtering strategies usually include several general steps and also specific steps according to individual panel settings determined by the validation process. Firstly, variants may be filtered by “variant type”, where intronic, synonymous, non-coding variants are often of little interest and considered non-pathogenic. Secondly, a VAF cutoff, under which variants can no longer be reliably distinguished from the background noise (assay detection limit), has to be made, all the while considering the absolute depth and the number of alternative reads (i.e., a VAF of 5% at 1,000x coverage is different to VAF of 5% at 20x coverage). Finally, remaining variants must be inspected in the context of polymorphism database (dbSNP), mutation databases (e.g., COSMIC, IARC TP53) and population databases (e.g., 1000 Genomes, GnomAD) to help differentiate between mutations and common polymorphisms. In silico prediction of functional impact should be used only as a supplementary tool for variant interpretation and never as the sole evidence for possible pathogenic impact (Li et al., 2017). After prioritization, variants with VAF of about 50% or 100% represent candidates for the investigation of their potential germline origin.

High confident clinically relevant somatic variants may be visualized using Integrative Genomics Viewer (IGV) (Thorvaldsdottir, Robinson & Mesirov, 2013). It helps to identify false positive variants, which were not filtered out during the bioinformatic and prioritization steps. Manually identified artifacts are often variants: (1) with low-quality base calls, (2) from the erroneous end of reads, (3) arising from the misleading alignment of repetitive or homologous regions, and (4) with strand bias.

#### CNV analysis in targeted sequencing

Chromosomal aberrations play an indisputable role in cancer development and generally contribute to human genome variation (Lupski, 2015). Targeted identification of deletions and amplifications of specific genomic loci in cancer patients acquired relevance after determining their evident or potential clinical impact (Concolino et al., 2018; Baliakas et al., 2019; Yu et al., 2020). In general, all types of CNVs can be detected by one or more bioinformatic methods (Zhao et al., 2013) including: (1) paired-end mapping approach, (2) split read-based approach, (3) read depth-based approach, (4) de novo read assembly of CNV events, or (5) a combination of these approaches. The most widely-used approach is based on split reads and is used to detect and localize breakpoints of any type of CNVs.

**Figure 3:**
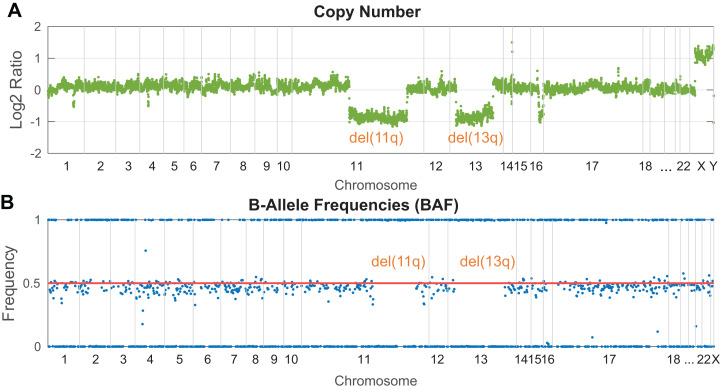
Visualization of clinically relevant CNV markers (del11q and del13p) detected in a peripheral blood sample from a patient with chronic lymphocytic leukemia. (A) Read depth approach, (B) B-allele frequency of analyzed SNPs. Probes along the chromosomes (*X*-axis) are depicted equidistantly.

In NGS targeted panels, a read depth-based strategy for CNV detection is employed in the majority of the developed software. This approach operates under the basic assumption that the number of DNA copies is proportional to the sequencing read depth in an analyzed genomic locus. Practically, the identification of copy number changes is based on the calculation of read depth variance between the tumor and normal sample within a given region. Such normalization helps remove biases arising from GC rich regions and non-uniformly covered target regions. However, a comprehensive normalization step is more challenging for samples with fragmented DNA (FFPE samples or cfDNA), where the signal is non-uniformly dispersed. The presence of a heterozygous deletion in the tumor sample leads to halved coverage in the affected region compared to the normal sample, while coverage in the unaffected regions remains approximately the same. Vice versa, a gain of chromosomal material results in increased read depth. A final plot of log2 normalized read depth displays the occurrence of known clinically significant CNV markers. Illustratively, Fig. 3 shows the identification of del(13p) and del(11q) in a patient with chronic lymphocytic leukemia. Conversely, it could be challenging to identify novel potentially relevant disease markers due to probe density and plot resolution given by assay design. Notably, shorter aberrations or minor clones can be barely visible in a noisy background. Circular Binary Segmentation (CBS) segmentation (Olshen et al., 2004) is used to translate and join these noisy copy number neutral or aberrant regions to segments, which represent an equal copy number state.

In a tumor-only scenario, the selection of an appropriate normal sample represents an additional task, which dramatically influences the precision of results. Two main approaches can be applied. Firstly, a copy number neutral reference sample sequenced in the same batch with the tumor samples can be used for normalization to ensure coverage uniformity. The most suitable material seems to be commercially available reference genomic DNA provided intentionally for human genome sequencing (Zook et al., 2016). Secondly, a “virtual normal sample” can be generated from an overall read depth mean of multiple tumor samples analyzed concurrently in a sequencing run. This statistical solution is implemented in software such as ExomeDepth (Plagnol et al., 2012), ONCOCNV (Boeva et al., 2014), CNVkit (Talevich et al., 2016) and panelcn.MOPS (Povysil et al., 2017). While ExomeDepth was designed to analyze CNVs in whole-exome data, ONCOCNV, panelcn.MOPS and CNVkit allow efficient analysis of targeted panels as well (Paradiso et al., 2018). Moreover, CNVPanelizer (Oliveira & Wolf, 2019) was explicitly developed to analyze CNVs in targeted panel assays. GATK’s best practice provides a pipeline for somatic CNV calling; however, this workflow was recently optimized for WES.

B-Allele Frequency (BAF) analysis constitutes a complementary method to identify copy number aberrations (Fig. 3B) with the benefit of copy neutral loss-of-heterozygosity (cnLOH) detection. BAFs are mostly seen in the context of SNP arrays and represent allelic frequencies of germline SNPs. Their values are expected to be 0 (SNP not present), 0.5 (a heterozygous SNP), 1 (homozygous SNP). In affected regions of tumor DNA, heterozygous SNPs are expected to be “out of phase”, shifted symmetrically above and below the heterozygous state. In perfectly pure tumor samples with fully clonal deletion, heterozygous SNPs in the affected region are expected to have BAF of 0 and 1. In reality, tumor samples are often “contaminated” by normal cells (especially in solid tumors) or contain minor subclones, which leads to the typical BAF range from <0 to 0.5> and <0.5 to 1> depending on the degree of contamination and the clonality of a given aberration. In targeted NGS, the informative strength and resolution of this approach are limited by the number of heterozygous SNPs analyzed in the target regions.

### A comprehensive analysis of genomic markers by the targeted custom NGS panel-practical experience from Czech laboratory

Finally, we would like to share our practical experience with the implementation of a versatile capture-based NGS panel targeting various molecular markers in lymphoproliferative diseases. We aimed to integrate analyses of several markers with established or potential prognostic and predictive impact in B-cell neoplasms, including gene mutations, chromosomal aberrations, immunoglobulin (IG) or T-cell receptor (TR) rearrangements, and clinically relevant translocations. None of the available commercial or published research panels (Rodríguez-Vicente, Díaz & Hernández-Rivas, 2013; Kluk et al., 2016; Hung et al., 2018; Kim et al., 2019) suited our needs, and therefore we decided for a capture-based technology utilizing UMIs and a custom design due to the advantage of tailored options. Finally, our panel with a total capture size of 1.13 Mb included probes for the analysis of: (1) all exons and splice sites of 70 protein-coding genes and all functional genes of IG and TR loci, (2) recurrent deletions 17p, 11q, 13q in the desired resolution (300 kb–1 Mb) and (3) genome-wide CNVs and cnLOH enabled by the evenly spaced backbone of probes. The identification of common translocations in lymphomas, that is t(11;14), t(14;18), t(3;14), was ensured by probes covering the whole IGHJ region.

The validation of experimental and bioinformatic procedures was performed by the sequencing of 63 DNA samples extracted from various types of biological material obtained from 49 patients with diverse lymphoproliferation. The validation sample cohort was selected to get a representative set of previously identified genetic alterations: (1) 109 SNV/Indels at various VAF (1–100%), (2) 79 CNVs (gains, losses) and cnLOHs of various extent (0.014–137 Mb) and tumor load (15–100%), (3) common translocations and IG/TR rearrangements. Commercial reference gDNA (NA24631; Coriell Institute, Camden, NJ, USA) was used in each sequencing run as a normal sample for CNV analysis and also for the assessment of panel performance (Hardwick, Deveson & Mercer, 2017). We validated several parameters according to available guidelines (Jennings et al., 2017) including accuracy metrics (positive percentage agreement, PPA; positive predictive value, PPV), sensitivity (limit of detection, LOD), reproducibility and specificity. The intended coverage was approximately 1000x after deduplication to achieve assay sensitivity of at least 5% VAF.

The in-house bioinformatic data analysis workflow consists of two independent branches: (1) a pipeline for detection of SNVs/Indels and CNVs implemented in Snakemake, and (2) a pipeline for the identification of IG/TR gene rearrangements and translocations. All scripts and software used in the first branch of bioinformatic analysis and with all non-default parameters are listed in Table S3. We measured the analysis run time (Fig. S1A) and memory (RAM) usage (Fig. S1B) in each step for ten samples analyzed in one sequencing run. Four CPUs cores with no parallelization were used, and the overall analysis running time was ~78 h. We will not dissect the second analytical branch since it exceeds the scope of this review.

Our panel demonstrated high coverage uniformity throughout all targets and across individual runs and showed the following basic parameters: (1) median coverage 921x after deduplication with 90% of targets >500x, 0.4% of targets <100x, (2) 43% PCR duplicates on average and (3) 27% off-targets reads on average (calculated before deduplication). The panel enables reproducible detection of SNV/Indels with high accuracy (PPA 100%, PPV 100%) and sensitivity when complying with our 5/5 rule for variant prioritization (i.e., VAF >5% and >5 variant reads) established during the validation process. The pre-defined LOD of 5% was corroborated by a dilution experiment. Manual inspection of suspicious variants in IGV was performed to avoid misinterpreting artifacts as true variants. The evaluation of CNV detection confirmed an expected high resolution of 300 kb–1 Mb in recurrently deleted loci of 17p, 11q and 13q arms and over 6 Mb across the whole genome depending on the backbone probe density. Out of all the tested CNVs, 91% were correctly identified. Seven undetected aberrations were below the resolution of the assay (either by extent or by their clonal proportion). The dilution experiment and validation results led us to set the threshold for CNV detection to log2 ratio ≥±0.2 and BAF ≥±0.1, which corresponds to the presence of at least 20% of cells with a respective chromosomal aberration in a sample. It is advantageous to combine both approaches, read depth and SNP analysis, since altogether, they provide complementary results.

In summary, we successfully implemented a versatile NGS capture-based tool for integrated analysis of molecular markers with research and clinical merit for patients with lymphoid malignancies (publication is under revision).

## Conclusions

The purpose of this review was to provide a guidebook for the development of a robust bioinformatic pipeline for the analysis of clinically relevant molecular markers detected by targeted NGS panel intended for routine use. We present an overview of contemporary bioinformatic approaches for the analysis of genomic aberrations supported by an example of a successfully implemented comprehensive capture-based NGS panel. According to our experience, the most crucial steps for targeted NGS tool development are: (1) appropriate selection of validation cohort comprising plenitude of representative samples and diverse targets, (2) careful optimization and validation of the analytical pipeline based on state-of-the-art bioinformatic approaches to ensure high accuracy of the results and (3) robust software and hardware environment. The whole procedure of specific tool implementation is rather time-consuming but highly rewarding, especially when a custom assay with long-term use needs to be established.

## Supplemental Information

10.7717/peerj.10897/supp-1 Supplemental Information 1 An example of clinically relevant variants with important annotations obtained by VEP. Variants were identified by custom NGS panel in several CLL samples.

10.7717/peerj.10897/supp-2 Supplemental Information 2 Supporting sources used for efficient genomic variant annotation and interpretation.

10.7717/peerj.10897/supp-3 Supplemental Information 3 Software and parameters used in our established in-house bioinformatic pipeline for the analysis of genomic markers in lymphoproliferative disorders.

10.7717/peerj.10897/supp-4 Supplemental Information 4 The measurement of fundamental hardware parameters in each step of the in-house bioinformatic pipeline established for our custom NGS panel data analysis. (A) computational time and (B) maximal RAM consumption. Steps on *Y*-axis are arranged in sequential order. *For details about QC analysis see Table S3.

## Additional Information and Declarations

### Data Availability

The following information was supplied regarding data availability: There is no raw data or code because our article is a literature review.
