# Supplementary figures and images for: Bioinformatic strategies for the analysis of genomic aberrations detected by targeted NGS panels with clinical application
Source: PeerJ. 2021 Mar 31;9:e10897. doi: 10.7717/peerj.10897 (PMC8019320; doi:10.7717/peerj.10897)

A

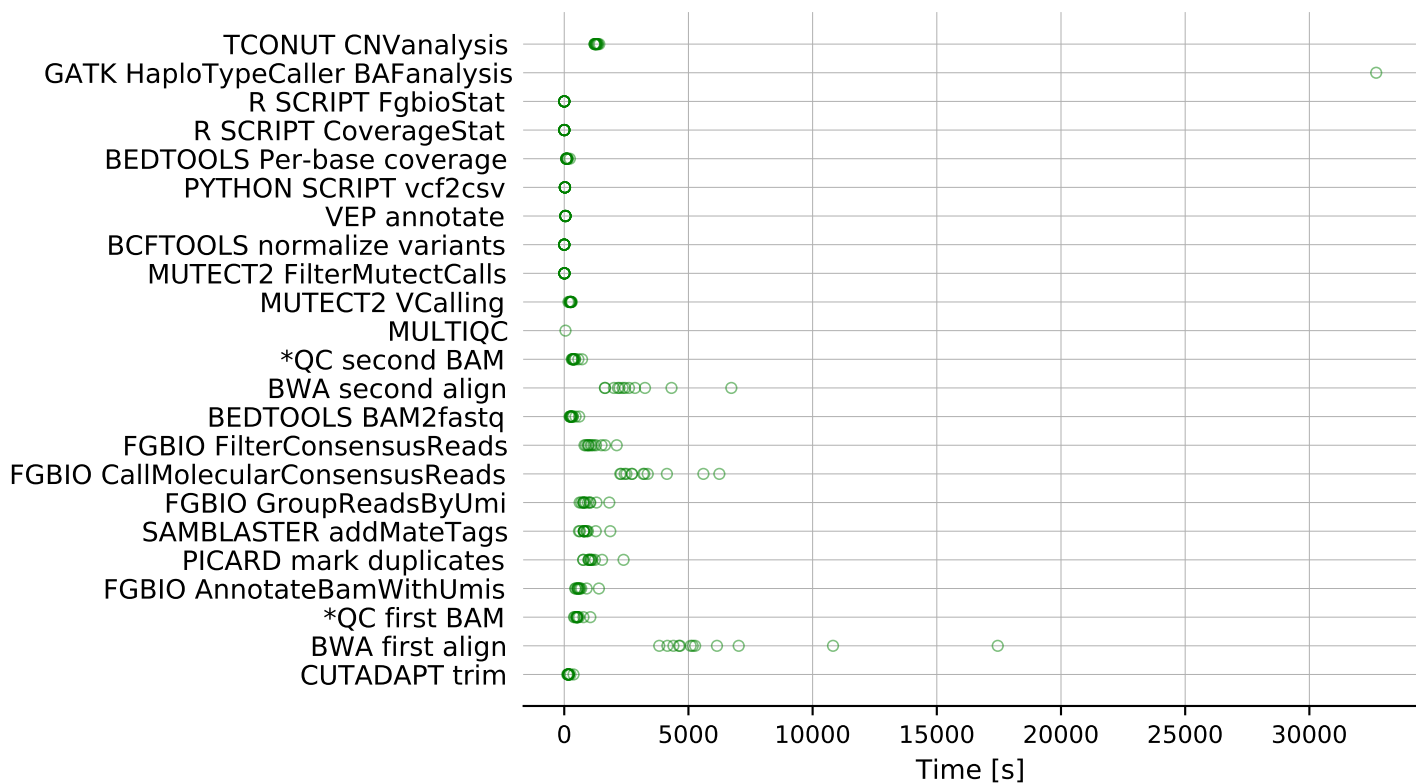

B

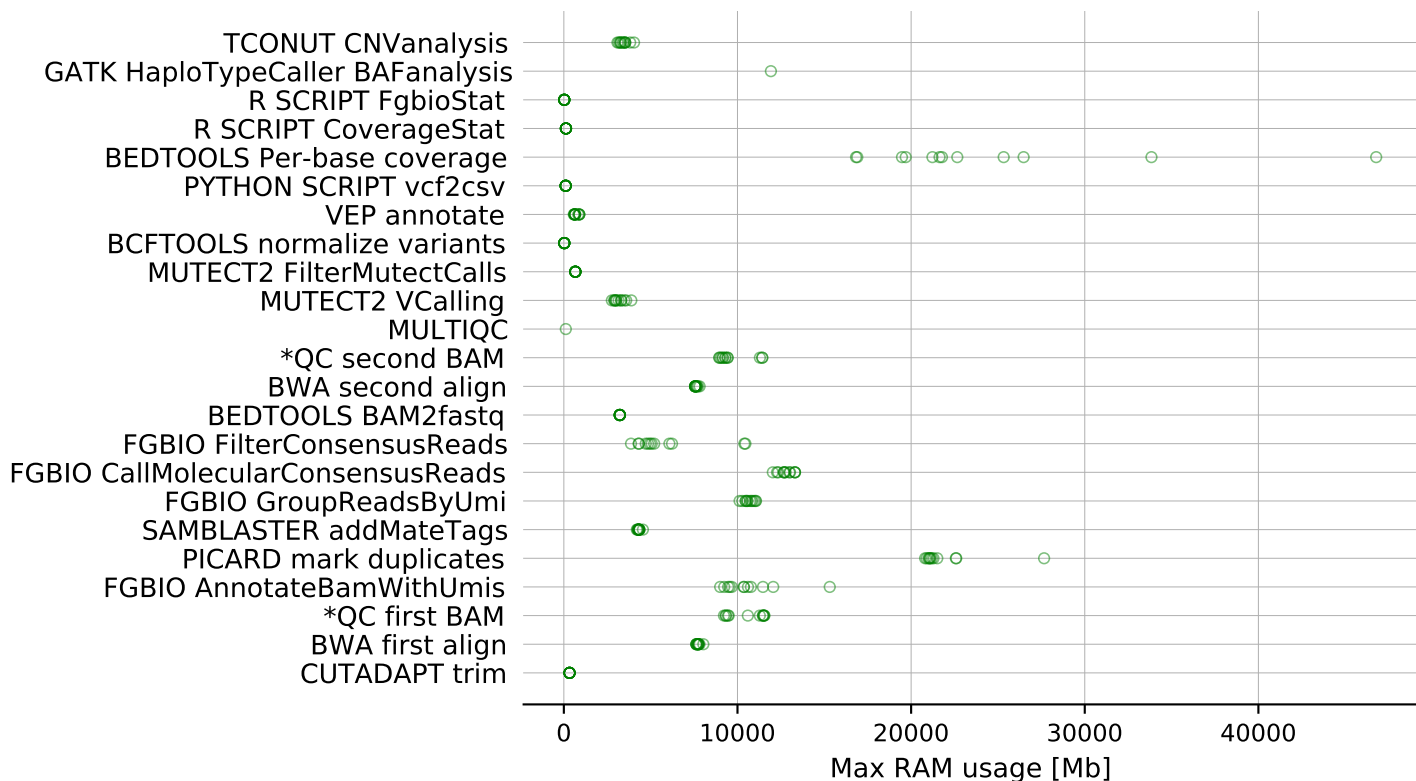

Supplement: Supplemental Information 4 — (A) computational time and (B) maximal RAM consumption. Steps on Y-axis are arranged in sequential order. *For details about QC analysis see Table S3. [file peerj-09-10897-s004.pdf]
